# Histogram-Based CRC for 3D-Aided Pose-Invariant Face Recognition

**DOI:** 10.3390/s19040759

**Published:** 2019-02-13

**Authors:** Liang Shi, Xiaoning Song, Tao Zhang, Yuquan Zhu

**Affiliations:** 1 The School of Computer and Communication Engineering, Jiangsu University, Zhenjiang 212000, China; jsjxy@just.edu.cn (L.S.); yqzhu@ujs.edu.cn (Y.Z.); 2 Jiangsu Province Engineering Laboratory of Pattern Recognition and Computational Intelligience, Jiangnan University, Wuxi 214122, China; taozhang@jiangnan.edu.cn

**Keywords:** collaborative representation-based classification, statistical histogram, 3D morphable face model, image recognition

## Abstract

Traditional Collaborative Representation-based Classification algorithms for face recognition (CRC) usually suffer from data uncertainty, especially if it includes various poses and illuminations. To address this issue, in this paper, we design a new CRC method using histogram statistical measurement (H-CRC) combined with a 3D morphable model (3DMM) for pose-invariant face classification. First, we fit a 3DMM to raw images in the dictionary to reconstruct the 3D shapes and textures. The fitting results are used to render numerous virtual samples of 2D images that are frontalized from arbitrary poses. In contrast to other distance-based evaluation algorithms for collaborative (or sparse) representation-based methods, the histogram information of all the generated 2D face images is subsequently exploited. Second, we use a histogram-based metric learning to evaluate the most similar neighbours of the test sample, which aims to obtain ideal result for pose-invariant face recognition using the designed histogram-based 3DMM model and online pruning strategy, forming a unified 3D-aided CRC framework. The proposed method achieves desirable classification results that are conducted on a set of well-known face databases, including ORL, Georgia Tech, FERET, FRGC, PIE and LFW.

## 1. Introduction

In recent years, with the huge development in computer sciences and informatics, representation-based methods (RBMs) have been given significant attention and garnered considerable achievements [1,2] in pattern recognition. The collaborative representation-based classification (CRC) method [3,4] is considered as the one of the most representative RBMs and has also been successfully applied in various research fields, including feature representation, image classification [5], machine learning and biometrics. Moreover, it has been proven that CRC has potential advantages in the applications of sparse representation [6,7] and low-rank representation [8].

In particular, the basic idea of CRC is to reconstruct a test sample using the dictionary set that includes all training samples across each subject [9]. The category is subsequently obtained by evaluating the collaborative capacity on the query face image across each subject and the test image is assigned to the class that has the minimum reconstruction error. Although CRC can effectively obtain the collaborative capacity from the dataset, it cannot handle the case in which the training samples of the same subject contain variations in illumination, expression and occlusion and lead to the huge difference between each other. In view of this kind of condition, Liu et al. [10] proposed a kernel CRC (KCRC) approach to improve the performance of SRC. A hierarchical collaborative representation-base classification(HCRC) [11] was proposed to extend representation-based method. It was proved that the accuracy of CRC can be improved substantially by minimizing not only the Euclidean distance between a query face and its approximator but also the Euclidean distances from the approximator to training faces in each class. Zhu et al. [12] performed CRC on patches and combined the recognition outputs of all patches to a designed multi-scale patch based collaborative representation for face recognition. Rodriguez [13] developed a discriminative dictionary by means of a framework of sparse representation-based method, which is applied to code the test sample for recognition task. A multiple-kernel sparse representation method for supervised and unsupervised learning was proposed by Thiagarajan et al. [14]. Meanwhile, aim to reduce computational cost in face occlusion problem, Yang [15] studied a Gabor occlusion dictionary. Cheng et al. [16] developed an $\ell_{1}$-graph for image classification, while Qiao et al. [17] constructed a subspace to reserve the $\ell_{1}$-graph for face recognition. In addition, Yanget et al. [18] synthesized a latent dictionary learning with SRC framework for face recognition. In [19], a method of assembling the similarity and distinctiveness of features into CRC was proposed by Yang et al. They also presented a more general model for classification. Liu et al. [20] improved the accuracy of CRC by evaluating the reconstruction error of the test sample. Recently, Xu et al. [21,22,23,24] proposed a series of simple-to-achieve sets of synthesized training samples for the goal of augmenting training set. Due to the fact that the traditional dictionaries built by raw images cannot fully represent the appearance of test samples, especially in the case of pose variations, more recently, deep learning has also been widely used for robust face recognition across pose variations and shown very promising results [25,26,27].

From the above-mentioned studies, we can discover that the ability of RBMs to uncover information of discrimination depends on the process of collaborative (sparse)-constrained signal reconstruction. An effective dictionary must be that it is preserving most important properties of signal with various types. Nevertheless, these algorithms can fail to exploit the valuable properties of signals with various types; this mainly results from redundancy and uncertainty issues, which lead to incorrect classification results. The rationale of the representation-based method can be summarized as follows: it is the fact the test sample with high correlation to training samples from the subjects of a dictionary will be quite reasonable to classify it into the class with high collaborative representation ability [28].

Despite extensive studies on the representation-based classification model, less attention has been focused on the evaluation of the histogram statistical metric information from the synthesized images generated by 3DMM for the purposes of competitive training data selection and pose-invariant face classification. In this paper, we focus on a new CRC method that comprehensively combines the merits from both the 3D morphable face model and the pruning strategy under the histogram statistical model. The proposed method provides three main contributions:

- We use fitting process of 3DMM to reconstruct the 3D shape and texture for 2D face images. By this means, we can synthesize a lot of virtual 2D face samples whose poses are frontalized from the arbitrary poses.
- We reduce the redundancy and uncertainty of the synthesized dictionary by designing a pruning strategy. The histogram statistical metric information of all the generated 2D face images is subsequently exploited and evaluated. We then discard the training images with small contributions to represent a test sample.
- We propose a complete H-CRC framework to perform pose-invariant face recognition by discovering the most representative training samples from the synthesized dictionary using 3DMM generated faces and the histogram-based metric learning.

Compared with the conventional CRC algorithm, the proposed method can be considered as an evaluation and pruning strategy under the H-CRC framework. Among the different variation types of face images, pose-invariant face classification is one of the most challenging tasks. To this end, a great number of algorithms have been explored during the past decades, including deep learning-based methods which can achieve amazing performance with a huge training face dataset and a well-designed CNN model. However, the training of a deep neural network usually require a huge amount of training data and expensive GPU devices. For deep learning-based approaches, we have already made some studies and compared them with the 3DMM-based approach. As shown in our previous studies [29,30], the performance of DNN-based approaches decreases rapidly as the degree of pose variation increases, especially when the head rotation is larger than 30 degree. In addition, for smaller pose variations such as 0${}^{\circ }$ and 15${}^{\circ }$, 3DMM-based approach also obtained competitive results as compared with DNN-based approaches. In this paper, we explored the use of a 3DMM in generating virtual training samples, as well as design of a histogram-based pruning strategy to achieve the effective statistical information of all the generated 2D face images, for the robust pose-invariant CRC-based face classification. It should be noted that the proposed method is based on Non-deep-learning-based strategy, which might not continuously outperform the deep learning-based method which is driven by a huge training data. But the designed histogram-based CRC using 3DMM in this paper can swiftly address the pose-invariant issue especially in the case of under-sampled face recognition.

The remainder of this paper is organized as follows: Section 2 briefly outlines the CRC method, which serves as a background to the proposed method detailed in Section 3. Section 4 offers analytical elaborations of the proposed method. Section 5 reports comprehensive experimental results obtained on several well-known face databases. Finally, concluding comments are presented in Section 6.

## 2. Outline of CRC

Input a K×M training samples set $\left\{x_{1,1},\ldots ,x_{K,M}\right\}$ to build a dictionary. Where *K* denotes the number of classes and *M* stands for the number of samples in each subject, a test sample $y\in R^{P}$ can be approximated by the linear combination of all the training samples:

(1) $$y\approx \sum_{m=1}^{M}\sum_{k=1}^{K}\alpha_{k,m}x_{k,m},$$

where $\alpha_{k,m}$ is the entry of the coefficient vector corresponding to the *m*th training sample in the *k*th class $x_{k,m}\in R^{P}$ and *P* is the dimensionality of a training or test sample. The entry $\alpha_{k,m}$ indicates the response of the corresponding gallery set to represent the query sample y. Equation (1) can be compactly re-described as follows:

(2) $$\begin{array}{c} \min{∥\alpha ∥}_{2}\\ s.t.\;\;y=X\alpha . \end{array}$$

where $X=\left[x_{1,1},\ldots ,x_{K,M}\right]\in R^{P\times KM}$ denotes the dictionary matrix which contains all the training samples and $\alpha ={\left[\alpha_{1,1},\ldots ,\alpha_{KM}\right]}^{T}$ stands for the coefficient vector that is estimated by solving the $\ell_{2}$-norm minimization problem. The optimization of Equation (2) is a typical least square problem, and α can be achieved by

(3) $$\alpha ={\left(X^{T}X+\mu I\right)}^{-1}X^{T}y,$$

where μ and and I respectively denote a small positive constant and the identity matrix.

We can evaluate the propensity of the *k*th class to represent the test sample after obtaining the coefficient vector, as below:

(4) $$c_{k}=\sum_{m=1}^{M}\alpha_{k,m}x_{k,m},$$

where $c_{k}$ stands for the reconstructed signal of the test sample merely using the linear combination of training samples of the *k*th class. The reconstruction error for the test sample using the *k*th class can be obtained by:

(5) $$E{\left(y\right)}_{k}=∥y-c_{k}{∥}_{2}^{2},$$

and the label of the test sample y is estimated by

(6) $$Label\left(y\right)=\underset{k}{\mathrm{argmin}}\left\{E{\left(y\right)}_{k}\right\}.$$

## 3. The Proposed Method

**Figure 1:**
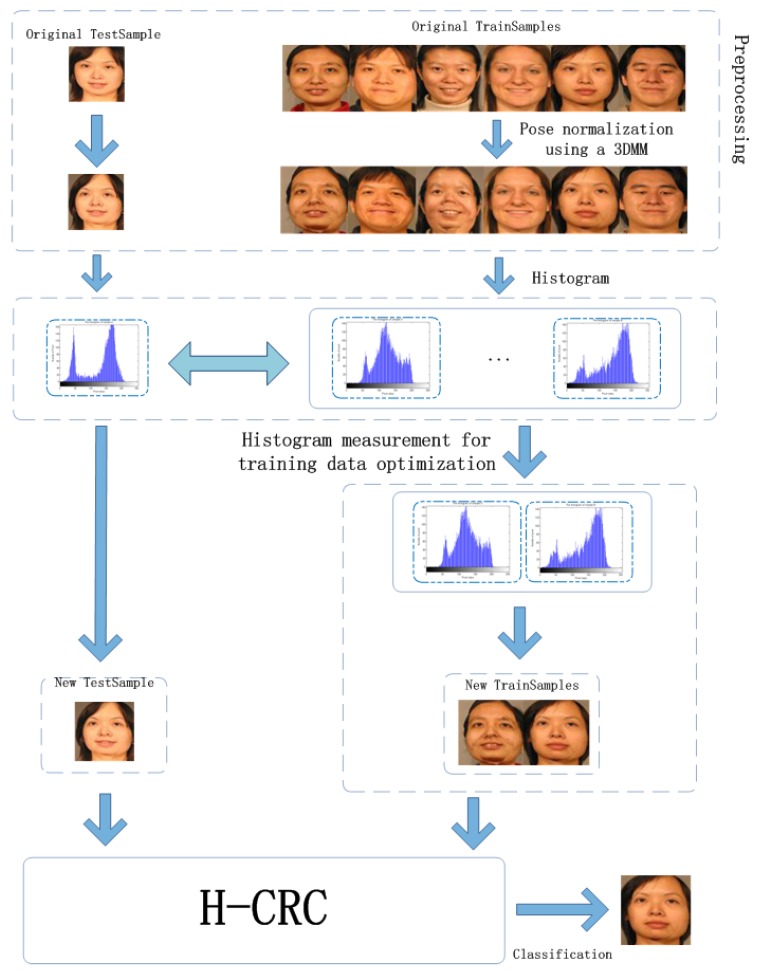
The schematic diagram of the proposed method.

As demonstrated in Section 2, although some improved representation-based classification methods can exploit important characteristics among complicated data sets, capturing certain possible variations in the statistical distribution information in each class space is still nontrivial [31]. Moreover, traditional methods cannot avoid the influence of data uncertainty effectively in a dataset in which the samples from subjects are informative and redundant. The classification performance may become compromised because of this condition. This motivates us to propose a novel histogram-based CRC (H-CRC) for 3D-aided pose-invariant face classification to evaluate the statistical distribution information across each subject. The schematic diagram of the designed algorithm is shown in Figure 1.

### 3.1. Pose Normalization by Use of 3DMM

First, we perform the pose normalization by means of a 3DMM that consists of a texture and a shape models. The texture and shape models are trained by projecting 3D shape (*x*, *y*, *z*) coordinates and associated texture (*r*, *g*, *b*) values onto two separate PCA spaces. Given an input image, 3DMM can recover the information of the input image, the shape, texture, camera (pose) and illumination parameters, via a fitting process. Once these parameters are estimated, the input face can be rendered in any given virtual view by adjusting the camera parameters. Note that the occluded pixels in the input image can be reconstructed using the estimated texture. For face recognition tasks, pose normalization is performed by converting a face under arbitrary poses to a frontal pose. Once the input image is fitted, pose normalization and frontal face rendering can be achieved by adjusting the camera parameters to a frontal view.

More specifically, a vertex $v={\left[x^{3d},y^{3d},z^{3d}\right]}^{T}\in R^{3}$ of a 3D shape is projected onto the 2D coordinates $s={\left[x^{2d},y^{2d}\right]}^{T}$ via a camera projection. We can divided the projection process into two parts: a rigid 3D transformation $T_{r}:R^{3}\to R^{3}$ and a perspective projection $T_{p}:R^{3}\to R^{2}$:

(7) $$\begin{array}{cc} T_{r} & :v^{\prime }=Rv+\tau , \end{array}$$

(8) $$\begin{array}{cc} T_{p}:s & =\left[\begin{array}{c} o_{x}+f\frac{v_{x}^{\prime }}{v_{z}^{\prime }}\\ o_{y}-f\frac{v_{y}^{\prime }}{v_{z}^{\prime }} \end{array}\right], \end{array}$$

in above formulation, $R\in R^{3\times 3}$ is the rotation matrix, $\tau \in R^{3}$ represents a spatial translation, ${\left[o_{x},o_{y}\right]}^{T}$ denotes the optical axis of the image-plane position, and *f* is the focal length.

**Figure 2:**
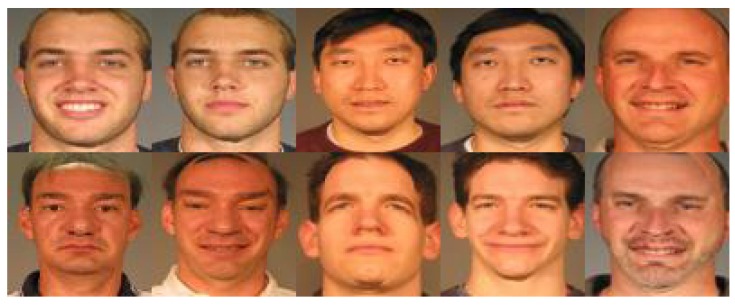
Some 2D face images rendered from input face images using a 3D morphable model (3DMM).

As a consequence, by setting different parameters of camera {R,τ,f}, the face image of any different pose can be rendered from the obtained 3D texture and shape. In this work, we perform frontalization; therefore, we only render an image to the frontal view. As shown in Figure 2, some 2D face images are rendered from an input sample using 3DMM.

### 3.2. Histogram Measurement for Training Data Optimization

From a probability prospective, the grey level of an input image can be viewed as the frequency of different grey scale pixel values. Thereby, the histogram corresponds to the probability density function p(r), and the probability distribution function P(r) represents an integral of the probability density function.

Assume that we have a discrete grey scale image *x*, and $n_{i}$ denotes the number of occurrences of grey level *i*. Thus, the probability of a grey level *i* in the picture can be obtained as follows:

(9) $$p_{r}\left(x\right)=p\left(r=x\right)=\frac{n_{i}}{n}\left(0\leq i<M\right)$$

where *M* denotes the total number of gray levels in the image (typical value is defined as 256), *n* stands for the total number of pixels of the image, and $p_{r}\left(i\right)$ is the histogram probability of each image for pixel value *i*, which is normalized to the range $\left[0,1\right].$ The probability density function $p_{r}$ and the probability distribution function P(r) can be obtained as:

(10) $$P\left(r\right)=\begin{array}{c} \int_{0}^{r}p\left(r\right)\;dr \end{array}$$

The histogram can be viewed as a one-dimensional column H, in which the *r*th element $h_{r}$ indicates the number of pixels corresponding to the luminance value.

(11) $$H=\left\{h_{0},h_{1},\ldots ,h_{255}\right\}$$

The second step of our method is to calculate the residual of the test sample’s histogram for each training sample by

(12) $$D_{k}\left(y\right)=∥H-H_{j}{∥}_{2}^{2}\left(j=1,2,\ldots ,m\right),$$

where *m* is the number of training samples; $H_{j}\left(j=1,2,\ldots ,m\right)$ denotes the corresponding histogram of each training sample $x_{j}\left(j=1,2,\ldots ,m\right)$; and H represents the histogram of the test sample. Thus, we can obtain *m* measurements between the test sample and each training database, that is, the evaluation of the histogram reconstruction is adopted for the whole training set. We then sort the achieved histogram measurements in ascending order as follows:

(13) $$\Omega =(d_{1},d_{2},\ldots ,d_{m})$$

By Equation (13), we choose the *L* best histogram measurements with smaller Euclidean distances to optimize the training set. Thus, the competitive training images $x_{1}^{\prime },x_{2}^{\prime },\ldots ,x_{L}^{\prime }$ that are closely associated with the test image are finally achieved. We then use these training samples that have the best representation capability to reconstruct the query sample and perform effective classification.

### 3.3. Perform Classification Using Obtained Nearest Neighbors

The third step of the designed H-CRC algorithm is to represent the test sample which can be described as a linear combination of the obtained *L* nearest neighbours samples. In this phase, the following equation can be approximately satisfied by:

(14) $$y=\beta_{1}x_{1}^{\prime }+\ldots +\beta_{L}x_{L}^{\prime },$$

where $x_{i}^{\prime }\left(i=1,2,\ldots ,L\right)$ stands for the identified *L* nearest neighbours and $\beta_{i}\left(i=1,2,\ldots ,L\right)$ denotes the coefficients. Equation (14) can be re-described as

(15) $$y=X^{\prime }\beta ,$$

where $\beta ={\left[\beta_{1},\ldots ,\beta_{L}\right]}^{T}$ and $X^{\prime }=\left[x_{1}^{\prime },\ldots ,x_{L}^{\prime }\right]$. We can solve β using $\beta =X^{\prime -1}y$ while $X^{\prime }$ is a nonsingular square matrix; otherwise, the coefficient β can be obtained by $\beta ={\left(X^{\prime }TX^{\prime }+\mu I\right)}^{-1}X^{\prime }Ty$, here, μ denotes a small positive constant and I stands for the identity matrix.

Considering that the nearest neighbours sample of test sample may be obtained from different subjects, we evaluate the sum of the contributions of these neighbours across each class for reconstruction of the test sample, we then assign the test sample to the class label that has the smallest reconstruction error. Concretely, if all the neighbours from the *k*th class (k=1,…,C) are $\xi_{s},\ldots ,\xi_{t}$, then the sum of the contribution that is measured for representing the test image of the *k*th subject is given as below:

(16) $$y_{k}=\omega_{s}\xi_{s}+\ldots +\omega_{t}\xi_{t}$$

The reconstruction error of $y_{k}$ from y is described as follows:

(17) $$D_{k}\left(y\right)=∥y-y_{k}{∥}_{2}^{2}$$

Evidently, the above formula allows the reconstruction error between the query sample and each $y_{k}$ to be evaluated in a fair way by measuring $∥y-y_{k}{∥}_{2}^{2}.$ We can conclude that a smaller deviation $D_{k}$ implies a greater contribution to representing the query image; and $y_{k}$ could be classified into the class that generates the smallest deviation.

### 3.4. The Detailed H-CRC Algorithm

The proposed H-CRC algorithm is described as Algorithm 1:

**Algorithm 1:** The Proposed H-CRC Algorithm.***1. input***: A dictionary that contains training set and a test sample y. ***2. preprocessing***: Perform the pose normalization using a 3D morphable model. ***3. for**i* = 1 to *N* (the number of test samples) ***do***
***4.*** Calculate the histogram residual of the test sample for each training sample by Equation (12). 
***5.*** Choose the best *L* histogram measurements with the smallest Euclidean distances for the test sample by Equation (13). 
***6.*** Represent the test sample using a linear combination of the obtained *L* nearest neighbors by Equation (14). 
***7.*** Evaluate the sum of the contributions of achieved neighbours across each class for representing the test image by Equation (16). 
***8.*** Perform the classification that produces the smallest reconstruction error by Equation (17). 
***End for***
***Return***: The final recognition rate.

## 4. Analysis of the Proposed Method

In this section, we will discuss about the characteristics, rationale and advantages of proposed method. The basic idea of our method is to develop a histogram-based CRC to generate a better representation of the test sample for 3D-aided pose-invariant face classification.

(1) *Histogram measures with pose normalization*

One of the key ideas of the proposed method is to perform the pose normalization using a 3DMM for converting a face under arbitrary poses to a frontal pose. According to the pixel distribution regularities for training images, the samples from the same subject should have similar pixel distributions. This motivates us to use a statistical histogram to intuitively describe the pixel-based features of the rendered image.

**Figure 3:**
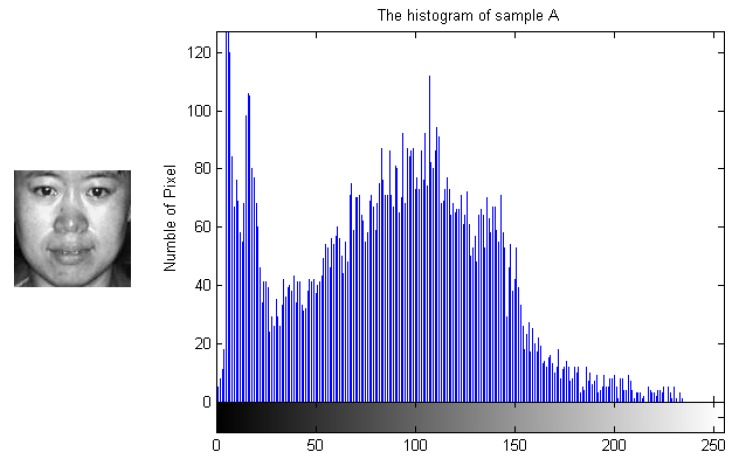
Sample A and its histogram.

**Figure 4:**
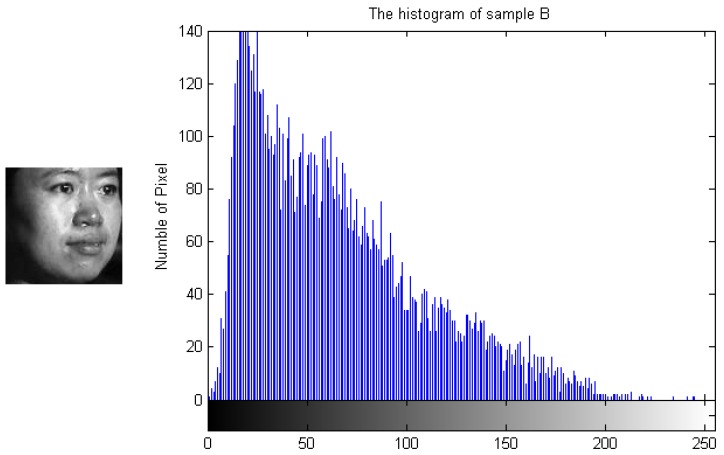
Sample B and its histogram.

**Figure 5:**
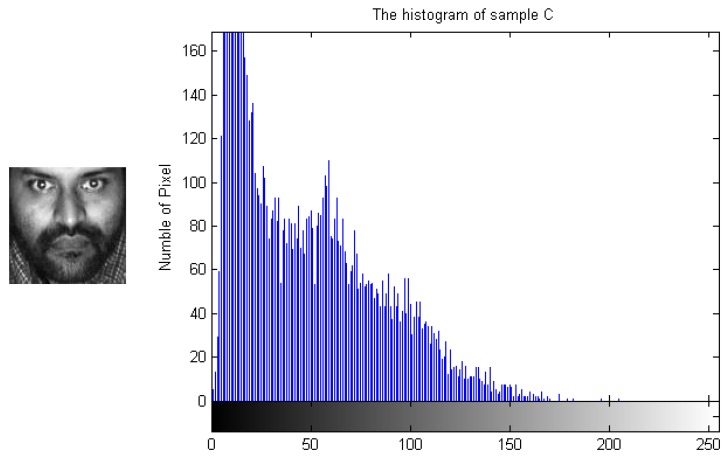
Sample C and its histogram.

**Figure 6:**
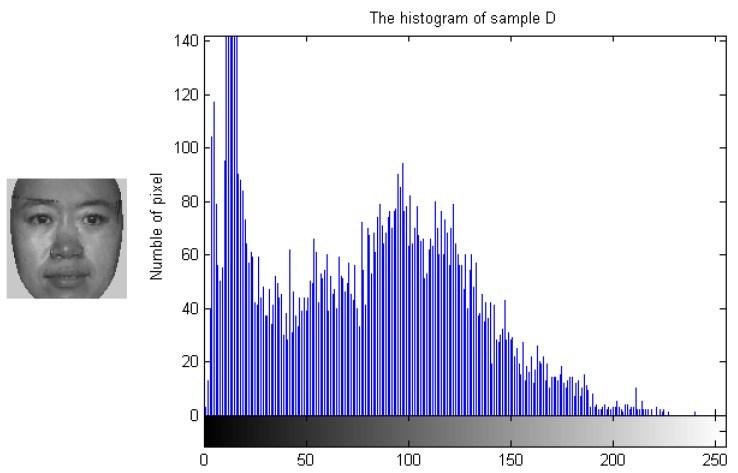
Sample D and its histogram.

To demonstrate how the designed algorithm works, Figure 3, Figure 4, Figure 5 and Figure 6 show the histogram difference among several face images evaluated on the ORL face dataset. Specifically, we randomly choose three training samples, A, B and C, to compare with their corresponding histograms. Note that images A and B are chosen from the same subject, sample C is selected from another subject. To demonstrate the merit of the proposed method, sample A represents a frontal face image, and sample B reflects a significant change in pose variations. As shown in Figure 3, Figure 4 and Figure 5, we note that the similarities of the pixel distributions of samples A and B are much lower than those of samples B and C; this easily leads to misclassification errors. Figure 6 shows the histogram of a synthesized image using a 3DMM to convert sample B with a large pose angle to a frontal view. Evidently, samples A and D have high similarity in their pixel distributions. Therefore, the pixel distribution information reflected by the histogram can be used to evaluate faulty training samples that adversely impact the representation-based classification.

(2) *The Basis of Histogram Measurement for Optimum Sample Selection*

In this method, we use a histogram to evaluate the pixel distribution of each sample for the purpose of training data pruning. In general, the range of the grey scale pixel values of the histogram is from 0 to 255, representing a total of 256 levels on the gray scale. Thus, a description of an image is transformed for grey scale analysis for the corresponding histogram. To judge the similarity of two images, measuring the Euclidean distance between them represents the most favourable method. Generally, the smaller the distance between two samples, the higher the similarity between the two samples.

As discussed in the previous section, the feasibility of employing histogram information is used to analyse the similarity between two samples. In this section, we will further discuss how we use the histogram measurement to evaluate the competitive samples. Specifically, the histogram of each image can be thought of as a one-dimensional column, where the *r*th element $h_{r}$ indicates the number of pixels corresponding to the luminance value. Thus, the evaluation of the similarity between two images can be easily transformed into the histogram measurement in terms of their variations in the pixel distributions of images. Meanwhile, in this method, an optimum selection scheme is presented for obtaining the competitive training samples.

**Figure 7:**
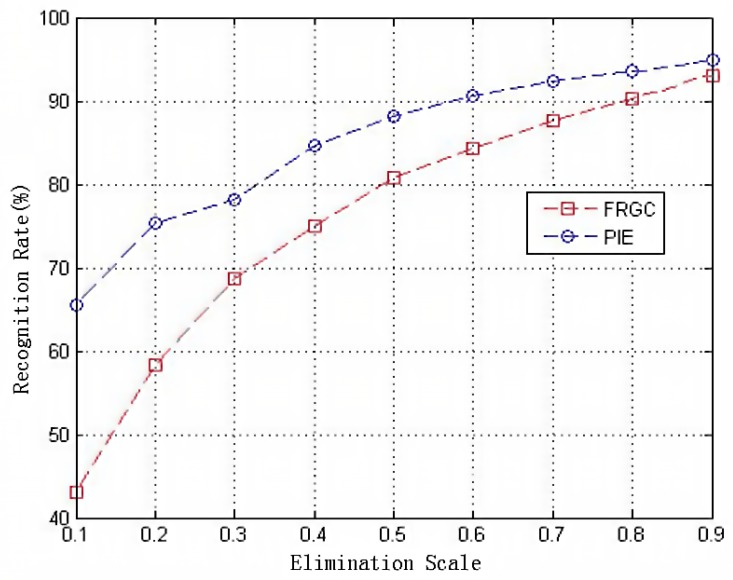
Classification results with different elimination scales of the training samples.

As shown in Figure 7, the proposed method gradually achieves better classification results on both the FRGC and PIE datasets, especially in the condition that the large portions of the training samples are eliminated. It is noted that the best classification result can be achieved under our method when using a 90% elimination proportion. Compared with other schemes for distance measurements, the histogram-based estimation can achieve a better tradeoff between time consumption and space complexity. The reasons for this are two fold. On the one hand, for a 256-dimensional vector, the complexity of the distance evaluation is tolerable. On the other hand, the statistics of all the pixel scale values can be used to reduce the loss of information when using histograms to perform training data pruning. To this end, we will prove our hypothesis via a specific instance that is detailed in the next section.

(3) *A Specific Instance*

**Figure 8:**
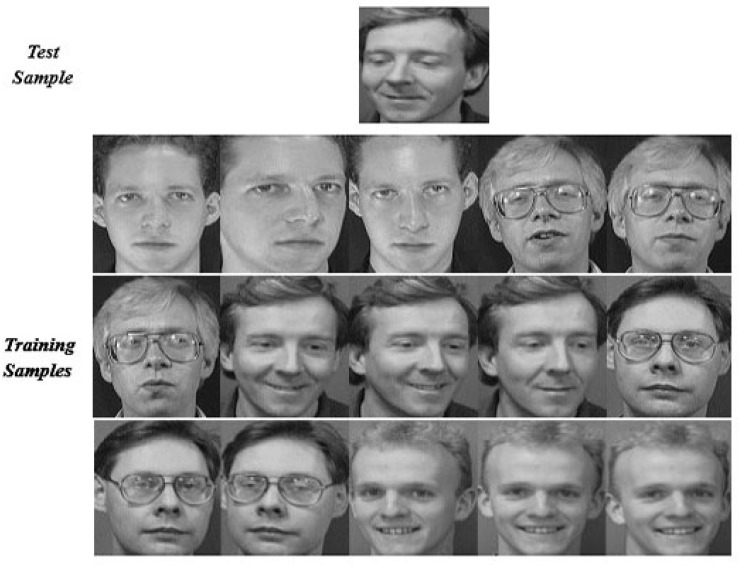
Some example faces of the ORL subset.

**Figure 9:**
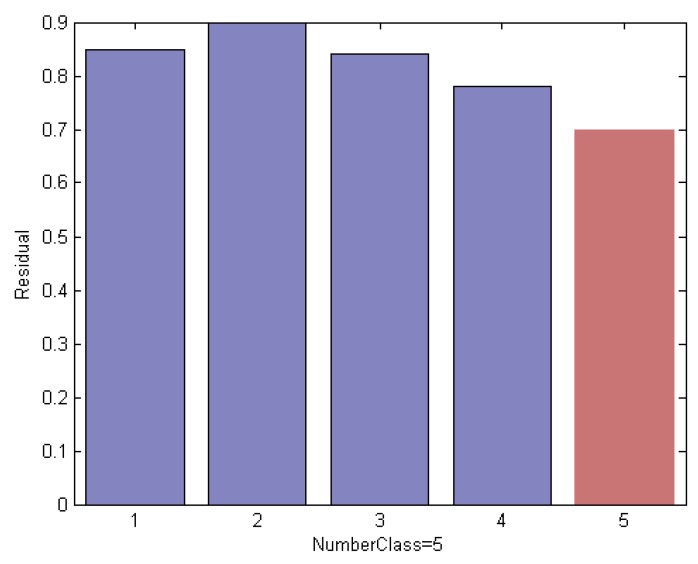
Reconstruction error of a test image using the original ORL subset, which leads to incorrect classification results.

**Figure 10:**
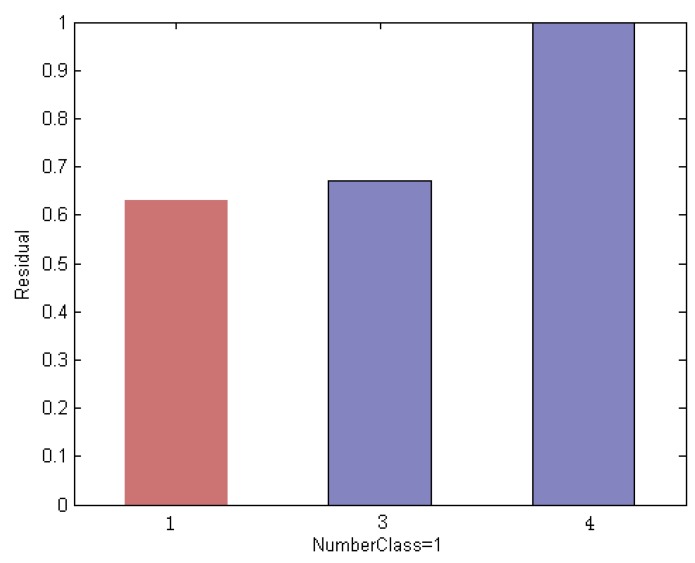
Reconstruction error of the test sample by each remaining class using an elimination scheme based on the Euclidean distance measurement, which still leads to incorrect classification results.

**Figure 11:**
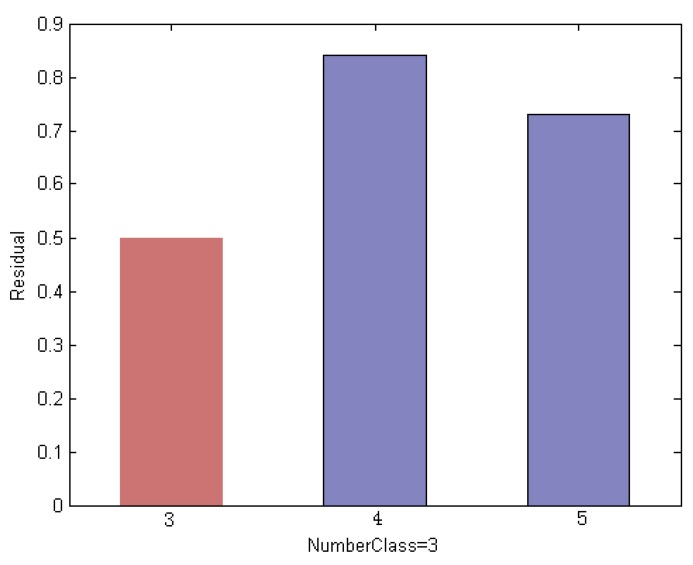
Histogram residual between the test sample and each remaining class obtained by histogram-based measurements. This shows that the test sample with the minimum reconstruction error is assigned to the label of the 3rd class, which leads to correct classification results.

In this section, we explain the proposed method by an empirical way. We evaluated on an ORL subset that includes the first 5 classes. We use the first 3 face images per subject for training and the remaining ones are used for test. Thus we created a training set of 15 images and a test set with 35 images. The example faces of this specific instance are shown in Figure 8; it is noted that the test sample is selected from the 3rd class of the subset. Using a different method, Figure 9, Figure 10 and Figure 11 indicate the comparison results of reconstruction error which is calculated by use of training samples of the respective class in the subset for representing a test sample.

Specifically, Figure 9 shows an example of a classification ordered by the residual degree between the test sample and each original training subject. We can see that the test sample reserving the minimum reconstruction error is assigned to the label of the 5th class, highlighted in red, which leads to incorrect classification results.

To reveal the nature of the designed method in this paper, the contribution of each subject to represent a query face image is measured in terms of residual. The subjects with the larger reconstruction error are eliminated from the original subset. Figure 10 shows the re-calculation of the reconstruction error of the test image by each remaining class using an elimination scheme based on the Euclidean distance measurement. Compared with the conventional method as shown in Figure 10, the residual of the test image by the 3rd subject is reduced when fewer classes in the original subset is used; however, the error remains slightly higher than that of the 1st class and leads to misclassification results.

The robust classification result is shown in Figure 11. The result is attributed to the histogram-based CRC mode, through which we can select competitive training samples to generate an optimum subset. Figure 11 shows the residual of the test image by each remaining class when using the histogram-based measurement. We note that the test sample with the minimum residual is assigned to the label of the 3rd class, which leads to correct classification results.

**Figure 12:**
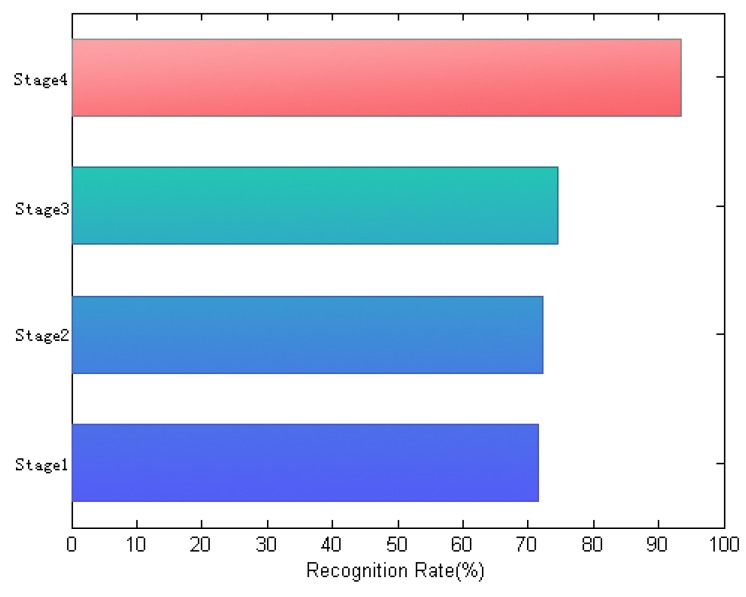
Different learning stages, denoted as Stage1: conventional collaborative representation-based classification (CRC), Stage2: histogram-based CRC, Stage3: histogram-based-elimination CRC and Stage4: histogram-based-elimination CRC using 3DMM.

Furthermore, to verify how the designed algorithm works, Figure 12 shows the recognition rates of different learning stages of the method, evaluated on a FRGC face subset that includes 100 persons with 30 different face images of each class. In this experiment, the first 10 training images per subject are selected as training samples, and the remaining 20 ones are used for test. As shown in Figure 12, the respective recognition rate is measured in terms of different dictionary learning stages, including conventional CRC, histogram-based CRC, histogram-based-elimination CRC and histogram-based-elimination CRC using 3DMM. From this experimental result, we can see that the joint combination of the histogram measurements and the pose normalization using 3DMM in our method can significantly improve the classification performance. In addition, the proposed method creates a dictionary that is learned from a dynamical optimization process, increasing the capacity of the representation to reconstruct input signals faithfully.

As discussed above, two aspects can give rise to the superiority of the proposed method. First, we generate the histogram-based measurement both from the test samples and the training set. Because the statistical histogram can be used to intuitively describe the pixel-based features of the images, a set of faulty training subjects with a less competitive representation capacity can be eliminated by this histogram-based metric learning. Second, we reconstruct the 3D shape and texture of each image by fitting 3DMM to raw iamges of 2D in the training set; thus, pose normalization and frontal face rendering can be achieved by adjusting the camera parameters to a frontal view.

## 5. Experimental Results

In this section, the comprehensive experimental results on a set of well-known face datasets are conducted, which includes ORL [32], FERET [33], GT [34], CMU-PIE [35], LFW [36], FRGC [37] and AR [38]. The facial images from these selected datasets were obtained with variations in illumination, expression and pose. Note that the proposed method is performed using the remaining competitive training samples that represent 10% of all samples that were frontalized by 3DMM.

**Figure 13:**
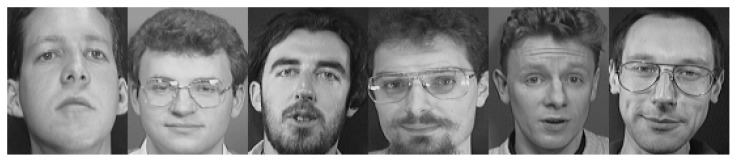
Sample images from the ORL face database.

The ORL face database [32] was collected by the Olivetti Research Laboratory in Cambridge. The dataset contains 40 distinct persons, and each subject includes 10 images. Those images were captured at different time period, with varying environment conditions, including facial illuminations, expressions and details. All individual images are in the upright and frontal position. Each image is resized to 56 by 46. Figure 13 shows a few image samples from ORL.

**Figure 14:**
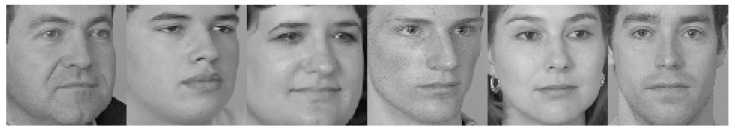
Sample images from the FERET face image database.

The FERET dataset [33] was created by the US Department of Defense through the DARPA program, which has become a benchmark database for the evaluation of face classification techniques. Our proposed algorithm was evaluated on a subset of FERET, which includes 1400 images of 200 individuals with seven different images of each person. We resized each face image in FERET to 40 by 40. Figure 14 shows a few image samples from FERET.

**Figure 15:**
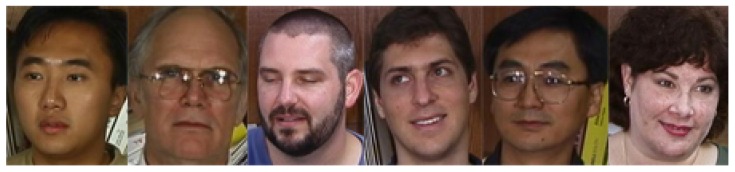
Sample images from the Georgia Tech face database.

The Georgia Tech (GT) face dataset [34] was produced by the Georgia Institute of Technology. This database includes 50 individuals which were taken over two or three sessions. All face images across each class in GT were captured by 15 colours with a cluttered background. The images show frontal and/or tilted faces with different illumination, expression and pose. We resized all of these images with different sizes to 40 by 30. Figure 15 a few image samples from GT.

**Figure 16:**
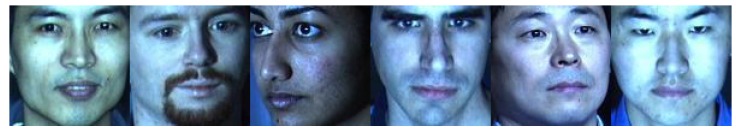
Sample images from the CMU-PIE face image database.

The CMU-PIE dataset [35] contains 41,368 images of 68 persons. All pictures in the dataset include mixed intraclass variations which are introduced based on 3 types of interference (each individual has 43 illuminations, 4 expressions and 13 poses). CMU-PIE has also become a benchmark database to evaluate face classification algorithms. In this paper, we perform our proposed method on a subset of CMU-PIE, including 6800 images of 68 persons with 100 different images (ten poses and ten illuminations) of each class. The images in the subset were resized to 100 by 100. Figure 16 shows a few image samples from CMU-PIE.

**Figure 17:**
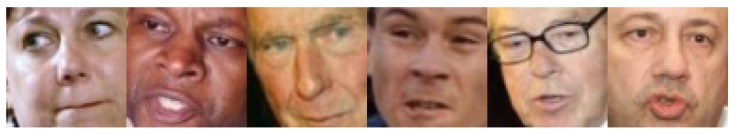
Sample images from the LFW face image database.

LFW [36] is considered as one of the most challenging datasets, which contains 13233 face images of 5749 persons of different gender, ages, etc. This face image dataset was characterized by abundant variations, including pose, illumination and expression variations. Our method is evaluated on a subset of LFW, including 1580 images of 158 persons with 10 different images of each class. Each face image in LFW subset was resized to 64 by 64. Figure 17 shows a few image samples from LFW.

**Figure 18:**
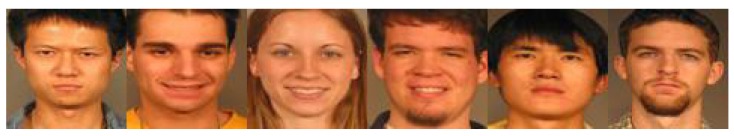
Sample images from the FRGC face image database.

The FRGC version 2 dataset [37] is viewed as a face recognition grand challenge database. It includes controlled and uncontrolled colour face images. The controlled images have good image quality, whereas the uncontrolled ones is with poor quality. All pictures of FRGC were taken under complex backgrounds. In this paper, we use 100 persons for experiments and each person contain 30 different face images. The pictures were resized to 80 by 80. Figure 18 shows a few images of FRGC.

**Figure 19:**
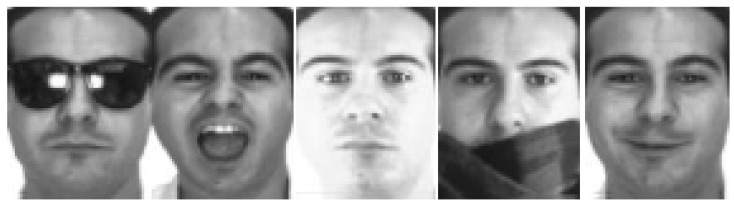
Sample images from the AR face image database.

The AR face database [38] contains about 4000 face images of 126 individuals, which consists of the frontal faces with different facial expressions, illuminations and disguises. There are two sessions and each session has 13 face images per subject. Figure 19 shows a few images of AR.

### 5.1. Results on ORL

We repeated our experiment 20 times and measured the recognition rate of different face classification algorithms on the ORL database. In each round of the experiment, θ(θ=2,3,4) training samples per subject were randomly selected for training, and the remaining ones were used for test.

The recognition rate of the proposed method is compared to sparse representation-based classification (SRC) [39], collaborative representation-based classification (CRC) [40], linear regression-based classification (LRC) [41], extended SRC (ESRC) [42], regularized robust coding (RRC) [43], relaxed collaborative representation (RCR) [19], TPTSR [44], SLC-ADL [45], Two-Step LSRC [46], DSRL2 [47] and other classical subspace-based classification methods, including Complete LDA (CLDA) [48], Locality Preserving Projection (LPP) [49], Marginal Fisher Analysis (MFA) [50], Orthogonal Laplacianface (OLPP) [51] and Supervised Orthogonal Discriminant Subspace Projection (SODSP) [52].

Table 1 shows the average recognition rate and standard deviation for each algorithm, regardless of the different numbers of training images involved. From Table 1, we can see that our method achieves much better results than other algorithms in terms of accuracy.

### 5.2. Results on FERET

For the FERET database, we also repeated our experiments 20 times. In each round of experiment, θ(θ=3,4,5) samples per subject were randomly chosen for training, and the remaining ones were used for test. Hence, a training set of 200×θ samples and a test set with 200×(7−θ) samples were generated.

We then used the histogram metric strategy to obtain the top 10 % competitive training samples from the synthesized dictionary using 3DMM for classification. The classification result of our method is compared to various sparse or collaborative representation-based algorithms, including LRC [41], CRC [40], ESRC [42], RCR [19], RRC [43], TPTSR [44], Two-Step LSRC [46], SLC-ADL [45], L1LS [53], DALM [54], Homotopy [55], FISTA [56] and DSRL2 [47].

As shown in Table 2, the proposed method consistently achieves much better classification results than the other methods, regardless of the different numbers of training samples involved.

### 5.3. Results on GT

For the GT database, we also repeated 20 times experiments and made comparison on different algorithms in terms of average recognition accuracy. We randomly select θ(θ=3,4,5) samples per subject for training, and used the remaining samples for test. Table 3 show the comparison of various methods, including LRC [41], CRC [40], ESRC [42], RCR [19], RRC [43], TPTSR [44], Two-Step LSRC [46], SLC-ADL [45], L1LS [53], DALM [54], Homotopy [55], FISTA [56] and DSRL2 [47].

From the experimental results of Table 3, we can draw a conclusion that our method achieves the best recognition rates of 68.88%, 72.69% and 79.88% to the corresponding θ(θ=3,4,5) training samples, which beats other classical sparse or collaborative representation-based classification methods.

### 5.4. Results on PIE

For the PIE dataset, the experiments were also repeated 20 times. In each round, θ(θ=5,10,15) images per subject were randomly chosen for training, and the remaining ones were used for test. Table 4 shows the recognition rates of different sparse representation-based methods, including LRC [41], CRC [40], ESRC [42], TPTSR [44], Two-Step LSRC [46], SLC-ADL [45] and the proposed method. According to these figures, we can see that the proposed method achieves much better classification performance than those of achieved by other methods across all different sizes of training sets.

### 5.5. Results on FRGC

For FRGC, we used a similar split as that on the PIE database to create the training and test sets. We demonstrated the experimental results of different methods including LRC [41], CRC [40], ESRC [42], TPTSR [44], Two-Step LSRC [46], SLC-ADL [45] and the proposed method in Table 5. It is noted that we also used same executed strategy as that on the above mentioned datasets and reported the average recognition rate as final result. As shown in Table 5, the proposed method achieves 79.96%, 93.45% and 95.47% recognition rates, regardless of different sizes of training samples involved, which are all better than those achieved by the other traditional methods. This is mainly because the collaborative representation is performed based on the histogram statistical metric information among the arbitrary pose variations.

### 5.6. Results on LFW

For the LFW database, we randomly selected θ(θ=1,2,3,4) images per subject as training samples, and used the remaining images for test. We also used same executed strategy as that on the above mentioned datasets. The experimental result of the proposed method is compared to LRC [41], CRC [40], ESRC [42], TPTSR [44], Two-Step LSRC [46] and SLC-ADL [45], which is shown in Table 6.

From Table 6, we can see that the proposed method beats the other algorithms. Noted that the performances achieved by the our method on LFW and FRGC are much better than those on the other databases. This is attributed to the fact that these two datasets contain more huge variations in appearance than the other databases. In this case, the advantage of our method is more evident compared to other algorithms.

The total of experimental results mentioned above demonstrate that the proposed method can achieve more effective and stable recognition accuracy than the other traditional methods, regardless of the numbers of training samples involved.

### 5.7. Comparison of Computation Time

**Figure 20:**
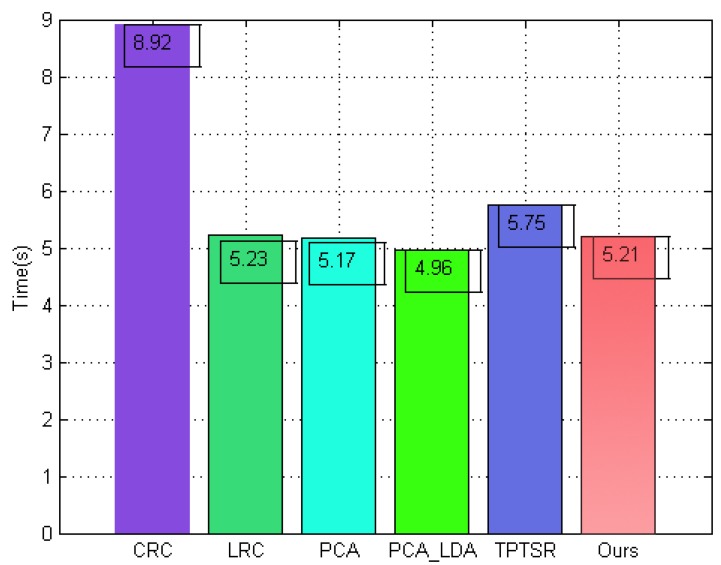
Running time of different methods.

To indicate the computational efficiency of the proposed method, we measured the performance of different face classification algorithms in terms of computation time, evaluated on ORL using the first 4 images of each subject as the training samples. The running time (in seconds) of our method is compared to the different sparse or collaborative representation-based methods, including CRC, LRC, ESRC, TPTSR, Two-Step LSRC, SLC-ADL, and other typical subspace-based classification methods such as PCA and CLDA. As shown in Figure 20, the proposed method consumes 5.21 s, which represents a better computational performance than other classical sparse or collaborative representation-based methods. Although the running time of our method is slightly higher than that of the classical PCA and CLDA algorithms, the acceptability of the computational complexity of our method in practice is entirely unaffected. Moreover, it should be noted that the computation process of our method is split into two stages. One step generates a number of virtual 2D face images, which are frontalized from the arbitrary pose variations; this step can be performed offline. The other step performs pose-invariant face classification using histogram statistical metric information and is completed online.

### 5.8. Experiment Comparisons with Some Deep Learning Based Methods

In this section, we compared the proposed method with some state-of-the-art deep models such as NN-CNN [57], VGG-SVM [58] and VGG-3DPD-CRC [4]. The experiments were conducted on some well-known challenging face datasets, including AR, LFW and FRGC. To be more specific, we first applied the VGG-FACE model to all the training and test images for robust facial features extraction, using computation of the forward propagation to obtain the convolution features. To make a comparison, the classical NN, SVM approaches and the recent 3DPD-CRC method have been used. The experiments were conducted on multiple face datasets. In practical applications, deep neural networks are able to extract robust features of images with appearance variations. With such robust features, even using a very simple classifier, such as the SVM classifier, can work well. It is worth noting that the proposed method does not aim at beating deep neural networks for robust feature extraction. In fact, the proposed algorithm should be treated as a more powerful sparse representation based classifier that can be jointly used with deep neural networks. For each subject of different dataset, a single image is randomly selected for training and the remaining ones are used for test. We repeated our experiment 10 times and measured the performance of different face classification algorithms in terms of recognition rate, which are reported in Table 7.

Moreover, in our method, using the histogram descriptor to replay raw images is a common and effective method to reduce data redundancy. If the raw image is not high-dimensional, the histogram with 256 bins is sufficient enough to effectively represent the original data. However, it might lead to a poor classification performance as the data size increases. To verify this notion, we used another partitioned histogram strategy [11] to enhance the face classification accuracy. To be more specific, we divided each image into 25 blocks and counted the histogram of each blocks. The final vector is jointed by all blocks to be the evaluation descriptor of dictionary pruning scheme. The results of SRC, CRC, NN-CNN, VGG-SVM, VGG-3DPD-CRC, the proposed VGG-H1-CRC using classical histogram descriptor and the enhanced version of VGG-H1-CRC, i.e., VGG-H2-CRC using partitioned histogram strategy are shown in Table 7. As shown in Table 7, the proposed VGG-H2-CRC beats all other methods in terms of face recognition accuracy across all different head rotations, including VGG-H1-CRC. This is attributed to the fact that the more robust collaborative representation is performed based on the 3DMM generated faces allied with the partitioned histogram-based pruning scheme.

### 5.9. Specific Experiment with Various Head Poses

To verify the effectiveness of the proposed method in pose variations, we designed an verified experiment to make sensitivity analysis across various head poses. To be more specific, the proposed H-CRC using VGG-FACE model, two traditional representation-based classification methods, i.e., SRC and CRC, and three deep learning-based methods, i.e., NN-CNN [57], VGG-SVM [58] and VGG-3DPD-CRC [4], were evaluated on a subset of PIE, for the reason that the PIE database contains much more variations in appearance than the others. The subset was chosen by 2992 images of 68 individuals with 11 pose variations, including 0${}^{\circ }$ (pose 06), ±22.5${}^{\circ }$ (pose 05, 07), ±45${}^{\circ }$(pose 04, 08), ±67.5${}^{\circ }$(pose 02, 03, 09, 10) and ±90${}^{\circ }$ (pose 01, 11) in yaw rotations, and 4 illumination variations per subject. We used 4 frontal images of each subject to generate the training dictionary and the remaining images were used for test. In the proposed method, we rendered 18 synthesized face images for each example to perform dictionary augmentation. The virtual face images were synthesized from ±10${}^{\circ }$ to ±90${}^{\circ }$ in yaw with the interval of 10${}^{\circ }$.

The comparison results of these two classical representation-based methods including SRC and CRC, as well as three deep learning-based methods, such as NN-CNN, VGG-SVM, VGG-3DPD-CRC and our VGG-H-CRC algorithm are shown in Table 8. According to this table, we can see that the proposed VGG-H-CRC method performs much better than the others in terms of face classification accuracy across all different head rotations.

## 6. Conclusions

In this paper, a novel histogram-based CRC for 3D-aided pose-invariant face recognition is developed. The designed method is aim to solve the under-sampled face classification problem including pose-invariant. The proposed method is designed to explore the use of a 3DMM in generating virtual training samples, as well as design of a histogram-based pruning strategy to achieve the effective statistical information of all the generated 2D face images, for the robust pose-invariant CRC-based face classification, which makes CRC robust in terms of face recognition rate while also being highly efficient in terms of computation time. We believe that our promising results can encourage future study on generating more meaningful pixel-based information for collaborative capabilities and better representation-based classification solutions.

## Figures and Tables

**Table 1:** Face recognition rates (%) of different methods on ORL database.

Method	Number of Training Samples		
	2	3	4
CLDA [48]	79.47 ± 3.11	83.39 ± 1.57	86.75 ± 2.42
LPP [49]	80.93 ± 2.71	87.78 ± 2.16	91.33 ± 1.42
MFA [50]	83.72 ± 3.27	91.42 ± 1.89	94.88 ± 0.98
OLPP [51]	84.31 ± 3.17	91.82 ± 2.09	95.50 ± 1.44
SODSP [52]	84.75 ± 3.06	92.61 ± 1.48	95.91 ± 0.93
CRC [40]	84.16 ± 2.47	90.65 ± 1.60	92.73 ± 0.93
LRC [41]	84.61 ± 2.08	90.29 ± 1.75	93.47 ± 1.22
SRC [39]	82.62 ± 2.07	88.74 ± 1.72	91.02 ± 1.39
ESRC [42]	83.08 ± 2.19	88.14 ± 1.94	93.08 ± 1.82
TPTSR [44]	83.12 ± 2.22	88.57 ± 1.66	93.75 ± 1.42
SLC-ADL [45]	71.87 ± 2.84	77.14 ± 2.18	83.33 ± 1.77
Two-Step LSRC [46]	85.32 ± 2.41	89.84 ± 2.06	94.18 ± 2.40
DSRL2 [47]	86.21 ± 2.13	89.71 ± 1.86	94.28 ± 1.47
Our method	**95.41 ± 2.05**	**98.14 ± 1.19**	**99.17 ± 0.85**

**Table 2:** Face recognition rates (%) of different methods on FERET database.

Method	Number of Training Samples		
	3	4	5
CRC [40]	50.41 ± 2.71	58.40 ± 2.57	64.50 ± 2.43
LRC [41]	69.78 ± 2.60	77.60 ± 2.46	81.12 ± 2.03
ESRC [42]	54.13 ± 2.56	71.33 ± 2.71	76.50 ± 2.36
RRC [43]	42.93 ± 2.84	53.74 ± 2.82	70.21 ± 2.63
RCR [19]	45.12 ± 2.70	51.02 ± 2.65	59.82 ± 2.48
TPTSR [44]	57.25 ± 2.53	65.12 ± 2.49	78.66 ± 2.15
SLC-ADL [45]	49.75 ± 3.06	68.33 ± 3.48	73.75 ± 2.93
Two-Step LSRC [46]	58.75 ± 2.67	77.13 ± 2.61	79.24 ± 2.90
L1LS [53]	59.43 ± 2.82	76.27 ± 2.77	82.63 ± 2.29
Homotopy [55]	54.14 ± 2.66	72.67 ± 2.56	77.45 ± 2.42
DALM [54]	59.79 ± 2.94	75.65 ± 2.80	80.91 ± 2.69
FISTA [56]	38.90 ± 2.99	50.54 ± 2.96	58.95 ± 2.92
DSRL2 [47]	61.69 ± 2.73	79.21 ± 2.46	81.16 ± 2.47
Our method	**86.91 ± 2.25**	**88.12 ± 2.41**	**88.77 ± 2.67**

**Table 3:** Face recognition rates (%) of different methods on GT database.

Method	Number of Training Samples		
	3	4	5
CRC [40]	46.62 ± 2.81	48.51 ± 2.76	51.75 ± 2.41
LRC [41]	53.53 ± 2.14	57.20 ± 2.57	60.22 ± 1.92
ESRC [42]	47.67 ± 2.02	51.64 ± 2.29	53.85 ± 2.37
RRC [43]	44.13 ± 2.82	43.44 ± 2.89	45.70 ± 2.74
RCR [19]	36.25 ± 2.87	37.89 ± 2.81	41.20 ± 2.84
TPTSR [44]	58.50 ± 2.62	65.82 ± 2.51	75.82 ± 2.15
SLC-ADL [45]	41.53 ± 3.13	49.09 ± 2.48	52.83 ± 3.04
Two-Step LSRC [46]	59.16 ± 2.18	67.81 ± 1.97	76.08 ± 1.93
L1LS [53]	51.40 ± 2.90	52.47 ± 2.79	61.66 ± 2.95
Homotopy [55]	45.74 ± 2.72	49.64 ± 2.76	52.46 ± 2.83
DALM [54]	52.75 ± 2.91	57.61 ± 2.84	61.30 ± 2.68
FISTA [56]	48.93 ± 3.02	51.99 ± 2.93	53.05 ± 2.99
DSRL2 [47]	54.12 ± 2.78	56.66 ± 2.72	61.82 ± 2.61
Our method	**68.88 ± 2.77**	**72.69 ± 2.80**	**79.88 ± 2.11**

**Table 4:** Face recognition rates (%) of different methods on PIE database.

Method	Number of Training Samples		
	5	10	15
CRC [40]	31.86	43.53	52.94
LRC [41]	31.08	49.41	52.65
ESRC [42]	33.29	50.92	54.76
TPTSR [44]	59.90	64.85	74.41
SLC-ADL [45]	28.82	43.02	45.29
Two-Step LSRC [46]	62.55	67.75	79.14
Our method	**82.35**	**91.29**	**93.82**

**Table 5:** Face recognition rates (%) of different methods on FRGC database.

Method	Number of Training Samples		
	5	10	15
CRC [40]	62.36	76.60	80.80
LRC [41]	40.52	54.35	62.73
ESRC [42]	66.54	79.07	82.93
TPTSR [44]	63.92	77.00	79.50
SLC-ADL [45]	36.29	48.53	59.91
Two-Step LSRC [46]	67.95	81.18	85.88
Our method	**79.96**	**93.45**	**95.47**

**Table 6:** Face recognition rates (%) of different methods on LFW database.

Method	Number of Training Samples			
	1	2	3	4
CRC [40]	8.34	11.58	14.03	15.63
LRC [41]	4.67	7.87	10.89	13.01
ESRC [42]	9.06	14.16	17.23	19.97
TPTSR [44]	10.09	17.79	19.59	23.81
SLC-ADL [45]	3.86	7.69	10.84	13.82
Two-Step LSRC [46]	11.17	18.35	21.42	25.40
Our method	**20.18**	**34.13**	**44.41**	**52.53**

**Table 7:** Face recognition rates (%) of deep learning-based approaches on different datasets.

Dataset	NN-CNN	VGG-SVM	VGG-3DPD-CRC	VGG-H1-CRC	VGG-H2-CRC
AR	97.29	97.63	98.86	99.12	**99.30**
LFW	54.11	54.92	60.24	62.73	**64.22**
FRGC	91.43	91.84	93.92	95.08	**96.13**

**Table 8:** Face recognition rates (%) with various head poses.

	Pose Number	01	02	03	04	05	07	08	09	10	11
Method											
SRC		2	5	4	7	18	22	7	4	7	5
CRC		3	5	5	7	18	21	8	5	8	4
NN-CNN		11	13	33	27	46	41	39	32	29	28
VGG-SVM		13	15	35	42	30	48	42	35	31	32
VGG-3DPD-CRC		33	34	58	49	69	59	51	41	38	39
VGG-H-CRC		**35**	**37**	**60**	**52**	**73**	**63**	**54**	**45**	**42**	**44**
